# Establishment of the circadian metabolic phenotype strategy in spontaneously hypertensive rats: a dynamic metabolomics study

**DOI:** 10.1186/s12967-020-02222-1

**Published:** 2020-01-28

**Authors:** Huanjun Wang, Xiaoming Wang, Dongmei Qi, Mengjia Sun, Qingqing Hou, Yunlun Li, Haiqiang Jiang

**Affiliations:** 1 School of Pharmaceutical Sciences, Shandong University of Traditional Chinese Medicine, Jinan, 250355 People’s Republic of China; 2 Experimental Center, Shandong University of Traditional Chinese Medicine, Jinan, 250355 People’s Republic of China; 3 Key Laboratory of Traditional Chinese Medicine Classical Theory, Ministry of Education, Shandong University of Traditional Chinese Medicine, Jinan, 250355 People’s Republic of China; 4 Shandong Provincial Key Laboratory of Traditional Chinese Medicine for Basic Research, Shandong University of Traditional Chinese Medicine, Jinan, 250355 People’s Republic of China; 5 TCM Clinical Research Base for Hypertension, Affiliated Hospital of Shandong University of Traditional Chinese Medicine, Jinan, 250011 People’s Republic of China

**Keywords:** Circadian rhythm, Hypertension, Metabolomics, Amino acids

## Abstract

**Background:**

Circadian rhythms play a fundamental role in the progression of cardiovascular events. Almost all cardiovascular diseases have a circadian misalignment usually characterized by changes in metabolites. This study aimed to dynamically monitor rhythmic biomarkers, to elucidate the metabolic pathways that are potentially under circadian control in spontaneously hypertensive rats (SHRs), and to eventually establish a circadian metabolic phenotype strategy based on metabolomics.

**Methods:**

In this study, an untargeted metabolomics technology was used to dynamically monitor changes in serum metabolites between SHR model group and WKY control group. Liquid chromatography-mass spectrometry (LC–MS) combined with multivariate statistical analysis was applied to identify markers of hypertension rhythm imbalance. The concentrations of amino acids and their metabolites identified as markers were quantified by a subsequent targeted metabolomics analysis. Overall, these approaches comprehensively explored the rhythm mechanism and established a circadian metabolic phenotype strategy.

**Results:**

The metabolic profile revealed a disorder in the diurnal metabolism pattern in SHRs. Moreover, multivariate statistical analysis revealed metabolic markers of rhythm homeostasis, such as arginine, proline, phenylalanine, citric acid, l-malic acid, succinic acid, etc., accompanied by an imbalance in hypertension. The key metabolic pathways related to rhythm imbalance in hypertension were found by enrichment analysis, including amino acid metabolism, and the tricarboxylic acid cycle (TCA). In addition, the quantitative analysis of amino acids and their metabolites showed that the changes in leucine, isoleucine, valine, taurine, serine, and glycine were the most obvious.

**Conclusions:**

In summary, this study illustrated the relationship between metabolites and the pathways across time on hypertension. These results may provide a theoretical basis for personalized treatment programmes and timing for hypertension.

## Background

Hypertension is a common cardiovascular disease characterized by a persistent increase in arterial blood pressure, which contributes to cardiovascular morbidity and mortality. Although hypertension has a high incidence in the population, there is no effective treatment for this disease; thus high blood pressure is known as a “silent killer” [1]. In recent years, hypertension, as one of the typical cardiovascular diseases, has been investigated in a series of studies using spontaneously hypertensive rats (SHRs) as model animals. Several biomarkers were previously found in a metabolomics study by this research group. These biomarkers were mainly associated with lipid, vitamin and amino acid metabolism [2, 3]. However, in these experimental studies of hypertension, changes in metabolites were measured at one time point, and dynamic monitoring was not performed. In addition, the circadian clock represents a key time-keeping mechanism for many fundamental physiological processes such as the cell cycle and metabolism [4]. As such, the determination of the mechanisms underlying hypertensive metabolic disorders at a fixed time is not a comprehensive approach. In general, the rhythmic oscillation of organisms naturally changes. Nearly all aspects of physical behaviours change over time, including light/dark, sleep/wakefulness, food intake/fasting, digestion, detoxication, and blood pressure [5, 6].

Animals possess an internal molecular mechanism called the ‘‘circadian clock’’, in which the endogenous, self-sustained oscillations over a period of approximately 24 h manifest in diverse physiological and metabolic processes [7]. Owing to metabolites oscillation throughout a day, clinical sampling at a single time point can easily lead to false or misleading diagnoses and even treatment outcomes [8, 9]. Circadian rhythms are generated by master clock neurons in the suprachiasmatic nucleus (SCN), which are critical in the physiology and behaviour of most organisms. The SCN and its efferent targets integrate light and feeding signals to entrain behavioural rhythms and to regulate clock cells in peripheral tissues, including the liver, adipose tissue and muscle [10]. Therefore, metabolic disorders are often associated with imbalanced circadian rhythms over time, resulting in many pathophysiological conditions such as obesity [11], metabolic syndrome, type 2 diabetes [12], and cancer [13]. The circadian rhythm is highly correlated with metabolites. The study of the circadian rhythm can reflect temporal changes in metabolites, which contributes to the comprehensive interpretation of a disease [6, 14]. To determine the underlying mechanism linking hypertension to metabolic disorders, it is essential to understand the temporal changes in differential metabolites, that is, the changes in metabolites under the influence of circadian rhythm. Previous research has found circadian rhythms of obesity, diabetes, hyperlipidaemia and other chronic diseases through metabolomics studies [11, 12, 14]; however, research on the rhythm of hypertension has not been reported. To discover rhythmic biomarkers and elucidate the metabolic pathways that are potentially under circadian control in SHRs, a circadian metabolic phenotype strategy based on metabolomics was established. In the present study, it was hypothesized that the metabolites associated with hypertension are abnormal within 1 day. Therefore, serum samples were collected from SHRs and Wistar-Kyoto Rats (WKYs) at multiple time points to compare metabolite changes. The entire research process was based on a liquid chromatography-mass spectrometry (LC–MS) metabolomics platform and amino acid analysis. By comparing the serum metabolites over a day, we found biomarkers and pathways associated with rhythmic changes in SHR model. Our results highlight the links between time and metabolites.

## Materials and methods

### Animals

All animal studies were approved by the Animal Ethics Committee of Shandong University of Traditional Chinese Medicine and all experimental procedures were performed in strict accordance with the international regulation of animal welfare. Nine-week-old male SHRs (215 ± 10 g) and WKYs (210 ± 10 g) were purchased from Beijing Vital River Laboratory Animal Technology Co, Ltd. (certificate of conformity: SCXK (Beijing) 2016-0006). Before the experiment, all the rats were adaptively fed for 1 week. The SHRs were considered the model group, and the WKYs were considered the control group. Six male rats from the model group and from the control group were randomly assigned to each time point (a total of 13 points) point maintained on a 12 h light/12 h dark cycle with standard chow and water provided ad libitum for 8 weeks. Afterwards, their blood pressure was measured by a BP-600 non-invasive tail artery blood pressure measurement system (Chengdu Techman Software Co., Ltd., Chengdu, China). Body weight was measured weekly. The volume of water and the weight of chow consumed by the rats in every cage were measured daily. Moreover, face temperature and pain threshold were also measured in the model and control groups by a SW-200 Photothermal Tail Pain Tester (Chengdu Techman Software Co., Ltd., Chengdu, China) and a GM300 Infrared Thermometer (Shenzhen Jumaoyuan Science and Technology Co., Ltd., Shenzhen, China), respectively. After 8 weeks, blood from the inferior vena cava was immediately collected after intraperitoneal injection of 1.5% sodium pentobarbital anaesthesia at a dose of 30 mg·kg^−1^.

### Chemical and materials

Acetonitrile (HPLC grade) was purchased from Fisher Scientific (Thermo Fisher, CA, USA); formic acid was purchased from Fisher Scientific (Thermo Fisher, CA, USA); water was obtained from Watson’s (Watsons Food and Beverage Co., Ltd., Guangzhou, China); and the amino acid mixture standard solution, type AN-II and type B, were purchased from Wako (Wako Pure Chemical Industries, Ltd., Japan).

### Untargeted analysis by UPLC-QE-MS

#### Preparation of serum samples

Blood samples were drawn into Eppendorf tubes and allowed to clot for 30 min before centrifugation. Then, the samples were centrifuged (1811×*g*, 15 min, 4 °C) to obtain serum samples. The serum samples were stored at − 80 °C until the metabolomics assay. For serum metabolite analysis, all samples were thawed at 4 °C. The serum samples (100 μL) and acetonitrile (400 μL) were mixed in a tube to remove proteins from the serum, and l, l-dichlorophenylalanine (60 μg/mL) was included as an internal standard. To ensure data quality and instrument stability during metabolic profiling, quality control (QC) samples were prepared by mixing the same volume of serum (20 μL) from 78 SHRs and 78 WKYs. The serum samples were shaken in a cryogenic oscillator for 5 min to mix evenly. Afterwards, the solution was placed at 4 °C for 15 min, and centrifuged at 15,294×*g* for 20 min. The supernatant was transferred into a clean 2 mL tube, dried with nitrogen and stored at − 20 °C. The samples were resolved with the initial mobile phase prior to LC-MS analysis.

#### Analysis of serum metabolites

Ultra performance liquid chromatography-electrospray ionization tandem mass spectrometry (UPLC-ESI–MS/MS) was performed using an electrostatic field orbitrap mass spectrometer (QE) coupled with an Ultimate 3000 UPLC System (Thermo Fisher, CA, USA). Instrument control, data acquisition and analysis were performed using Xcalibur software 3.0 (Thermo Fisher, CA, USA). The sample vials were maintained at 10 °C in a thermostatic auto sampler. Chromatographic separation was achieved on a Halo-C18 column (2.1 × 100 mm, 2.7 μm; AMT, USA) with the column temperature set to 45 °C. A total of 5 μL of each sample was injected into the column. The mobile phase consisted of solvent A (0.05% formic acid in water) and solvent B (0.05% formic acid in acetonitrile). The linear gradient programme was as follows: 0–1 min, 2% B; 3 min, 40% B; 15 min, 98% B; 17 min, 98% B; 17.1 min, 2% B; and 3.0 min of equilibration. The flow rate was 0.3 mL·min^−1^. Before running the samples, 6 QC samples were run to balance the instrument, and then 1 QC sample was run after every 6 samples. Meanwhile, the mass spectrometry detections were performed as follows: capillary temperature, 350 °C and spray voltage, 3.5 kV and 3.0 kV for positive ion mode and negative ion mode, respectively. The mass scan range was from 80 to 1200 Da. The mass resolution was set to 70,000.

#### Multivariate statistical analysis

The acquired mass spectrometry data (.raw) were exported into Compound Discoverer (CD, Thermo Fisher, CA, USA) software for data analysis. The CD software converts mass spectrometry data into metabolite information. These metabolic discoveries are achieved by using a combination of open online databases, local databases, and MS/MS metabolites data, which greatly improves the accuracy of metabolite identification. The software has the function of multivariate statistical analysis, including the peak area ratio between groups, log_2_FC and significance analysis. To identify the metabolic profiles of the control and model groups, unsupervised principal component analysis (PCA) and supervised partial least-square analysis (PLS-DA) were applied using SIMCA-P version 13.0 (Umetrics AB, Umea, Sweden). The p value and adjusted p value for multiple testing of each metabolite were determined by the CD software. Heatmap and pathway analysis were performed with MetaboAnalyst 4.0 software (http://www.metaboanalyst.ca).

#### Quantitative analysis of amino acids

Amino acid analysis was performed on a Hitachi l-8900 Amino Acid Analyzer with an EZChrom Elite system. Serum samples were thawed at 4 °C. A 100 μL serum sample was used for amino acid extraction that used 5-sulfosalicylic acid dehydrate to remove protein. The above sample was diluted with 200 μL of HCl (0.02 mol/L) and centrifuged at 15,294×*g* for 20 min before analysis. Chromatographic separation was performed on an ion exchange column. Mobile phases consisted of a buffer solution and ninhydrin. The standard sample was prepared according to the requirements and the amino acids in the serum were quantified by an external standard method.

#### Data analysis

The data are expressed as the mean ± standard deviation (SD, n = 6). Statistical significance was determined with SPSS 17.0 software (SPSS Inc., Chicago, IL, USA). Independent sample t-tests were used to assess significant differences. All p values were two-tailed, and p < 0.05 was considered to be significant.

## Results

### Baseline description

**Fig. 1:**
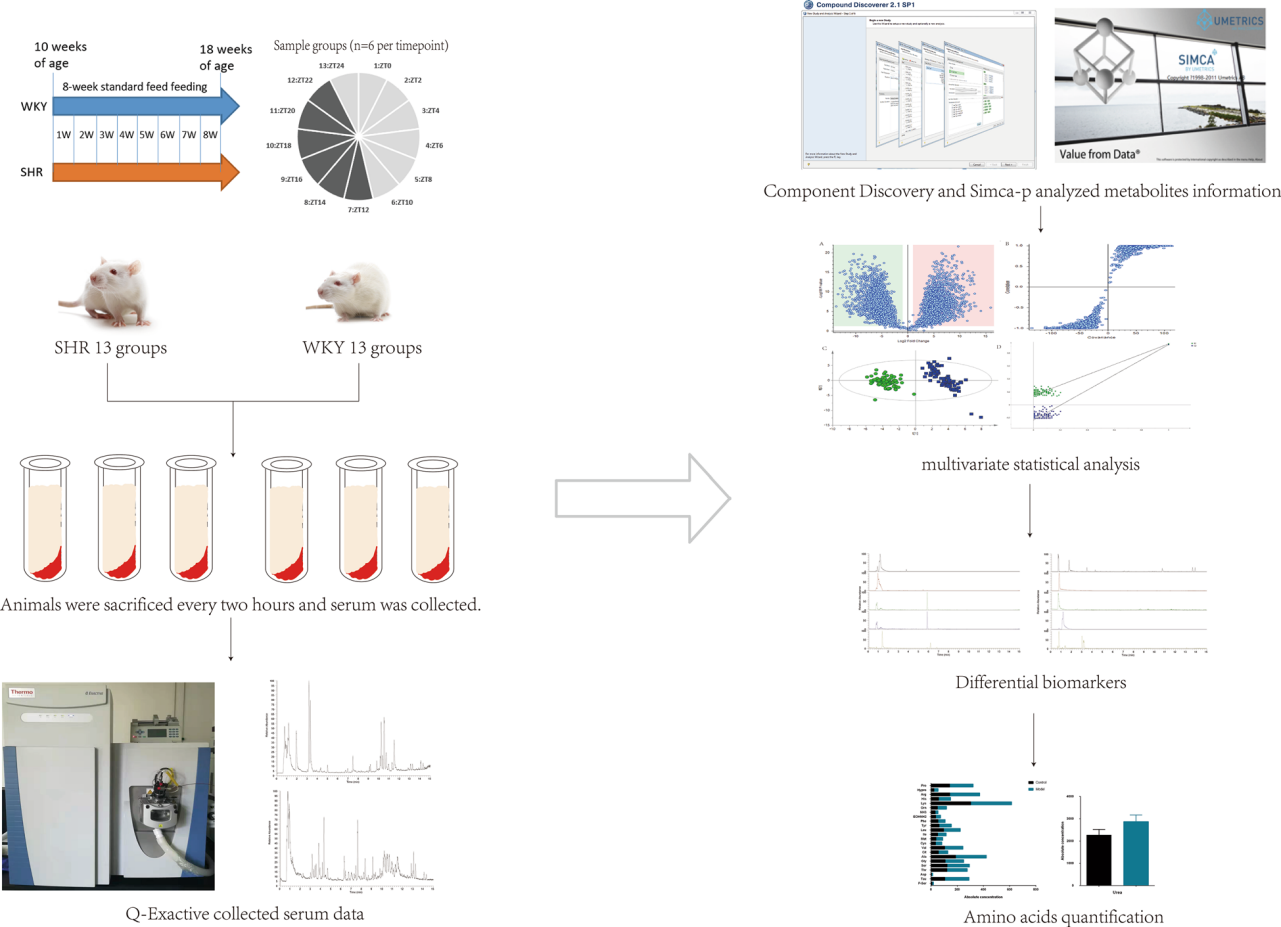
Flowchart of the whole study including the model groups and control groups. A total of 156 rats, including 78 SHRs and 78 WKYs, were divided into 13 groups. After 8 weeks, the rats were sacrificed every 2 h, and serum was collected. UPLC-Q-exactive-MS was used to collect serum data, and Compound Discoverer was used to analyse metabolites. The metabolites showing a circadian rhythm and associated with hypertension were regarded as differential biomarkers. Enrichment analysis of the biomarkers found that amino acid metabolism is an important rhythm-related metabolic process. Based on this result, amino acids were quantitatively analysed to determine the trend of each amino acid over 24 h

A total of 156 rats (78 SHRs and 78WKYs) were included in the study (Fig. 1). General characteristics, including blood pressure, body weight, water intake volume, food intake, face temperature and pain threshold, were shown in Table 1.

**Table 1:** General characteristics of WKYs and SHRs

	Control	Model
Body weight (g)	210 ± 10	215 ± 10
Blood pressure (mmHg)	111.22 ± 6.42	181.58 ± 5.25**
Food intake (g/d/cage)	100.06 ± 11.72	108.78 ± 13.25
Volume of drinking water (mL/d/cage)	192.27 ± 4.56	167.39 ± 12.49**
Face temperature (°C)	30.73 ± 0.29	31.26 ± 0.72
Threshold of pain (S)	7.89 ± 0.74	7.98 ± 0.25

** *p *< 0.01 the SHR model group compared with the WKY control group

To assess the difference in blood pressure and other indicators, the control group and model group were compared by *t* test. Table 1 shows that the blood pressure of the SHR group was significantly different from that of the control group. The body weight was not significantly different. According to the analysis of the volume of water consumed, the rats in the model group consumed much more water than did the rats in the control group (p < 0.01). However, the food intake pattern was the opposite of the water consumption pattern. The differences in the face temperature and pain threshold of the two groups were small and not significant.

### Establishment of the circadian metabolic phenotype strategy

**Fig. 2:**
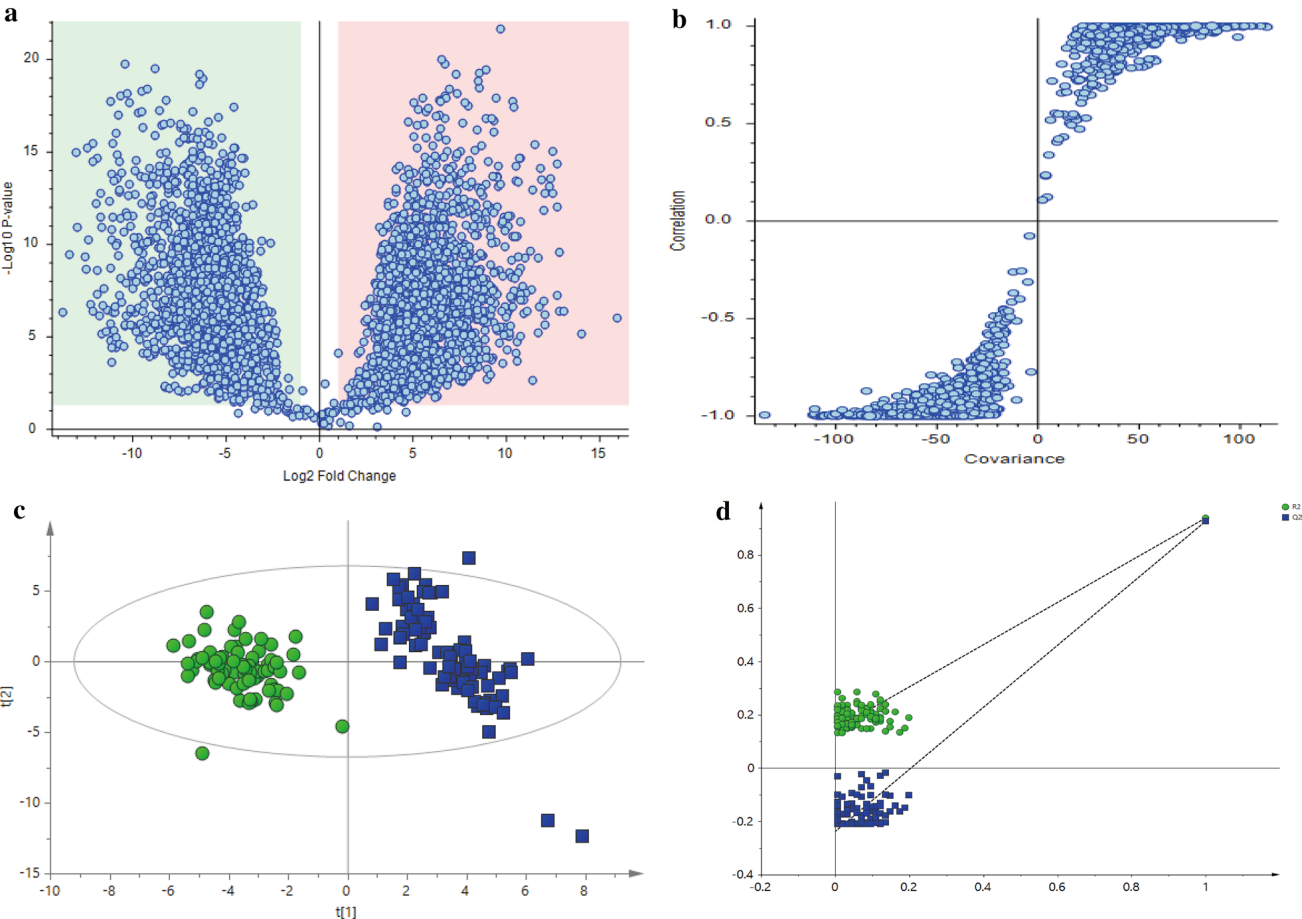
Log2 fold change (**a**), S-plot (**b**), and PLS-DA plot (**c**) showing 86 metabolites in the control group (green) and model group (blue); R2Y (cumulative), 0.894; Q2 (cumulative), 0.896. R2 is a measure of fit, i.e., how well the model fits the data. Q2 indicates how well the model predicts new data. A large Q2 (Q2 > 0.5) indicates good predictivity. A 100 permutations test (**d**) verified the accuracy of the model [R2 = 0.939, Q2 = 0.927]

Multivariate statistical analysis was used to assess the daily rhythmicity of the two groups. A total of 112 metabolites showed significant daily rhythms in the control group, and 86 metabolites were significantly different between the control group and the model group (p < 0.05). Among the 112 metabolites, four key metabolites (20%) in the tricarboxylic acid cycle (TCA) showed daily rhythms over 24 h, namely, succinic acid, l-malic acid, 2-oxoglutaric acid, and citric acid. Additionally, these metabolites were all included in the 86 differentially expressed metabolites. Ten metabolites (13%) in aminoacyl-tRNA biosynthesis pathway (75 metabolites in this pathway) were found in the control group, namely, l-histidine, l-phenylalanine, l-arginine, l-glutamine, l-valine, l-isoleucine, l-leucine, l-tyrosine, l-proline and l-glutamic acid. Nine metabolites in the arginine and proline metabolism pathways and seven metabolites in the pyrimidine metabolism pathway accounted for 16% of their metabolic pathways, including l-glutamine, ornithine, citrulline, l-arginine, l-glutamic acid, l-proline, 4-oxoproline, creatine, and creatinine in arginine and proline metabolism, and l-glutamine, uridine, cytidine, cytosine, thymidine, thymine, and pseudouridine in pyrimidine metabolism. This finding indicates that the circadian rhythm is significantly related to the TCA, aminoacyl-tRNA biosynthesis, arginine and proline metabolism, and pyrimidine metabolism. The metabolites with significant differences between the control group and model group were determined by log_2_FC analysis (Fig. 2a), S-plot analysis (Fig. 2b), and PLS-DA (Fig. 2c) [R2Y (cumulative), 0.894; Q2 (cumulative), 0.896]. PLS-DA showed a clear separation between the two groups in Fig. 2c. A 100 permutation test (Fig. 2d) was used to verify the accuracy of the model [R2 = 0.939, Q2 = 0.927].

**Fig. 3:**
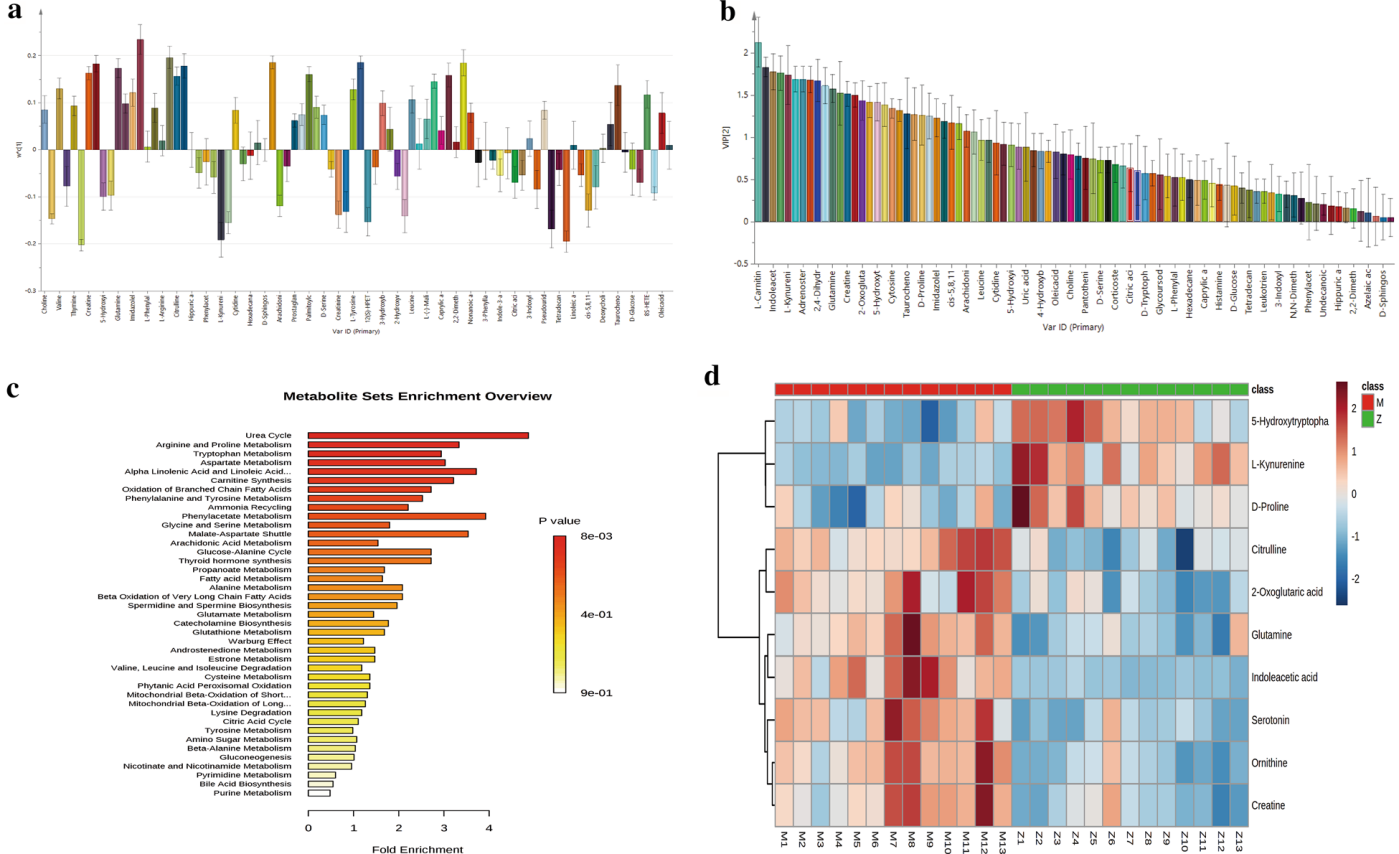
Loading plot (**a**) of the model groups and control groups, where the positive values represent metabolites with higher concentrations in the model group. The further away a point is from the x-axis, the greater the difference between the two groups. Every two metabolites are labelled on the x-axis. VIP plot (**b**) obtained by PLS-DA. The higher the VIP value is, the greater the contribution to the separation of the two groups. Enrichment analysis (**c**) of significantly differential metabolites. A higher position and deeper colour demonstrated a strong correlation between the enriched metabolic pathways. Heatmaps (**d**) of 13 groups (n = 6). A deep red colour represent high content, and a dark blue colour represent low content

The changes in these metabolites within the model group are shown in the loading plot (Fig. 3a). This plot indicates that mainly amino acids, serotonin, and adrenosterone were positively correlated with the model group and thus, positively correlated with hypertension, whereas glucose, proline, d-tryptophan and citric acid were negatively correlated with hypertension. To maximize the sampling differences between the study groups and to explore the most obvious metabolites with a circadian rhythm, metabolites with a VIP > 1 were selected (Fig. 3b). The VIP plot was obtained by PLS-DA. The higher the VIP value is, the greater the contribution of that metabolite to the separation of the two groups. Finally, 30 metabolites (36%) among the 86 metabolites showed significant differences between the two groups, and these metabolites allowed the two groups to separate well in PLS-DA. To further identify biomarkers with significant differences, the metabolic pathways were enriched by analysing markers with a VIP > 1 (Fig. 3c). The metabolic pathways with p < 0.05 were identified as differential metabolic pathways by enrichment analysis.As a result, three differential metabolic pathways were found under these conditions, including the urea cycle, arginine and proline metabolism, and tryptophan metabolism. The biomarkers associated with these differential metabolic pathways included ornithine, glutamine, indoleacetic acid, citrulline, serotonin, l-kynurenine, d-proline, 2-oxoglutaric acid, creatine, and 5-hydroxytryptophan. The expression levels of the abovementioned 10 metabolites in the two groups, as well as the changes over 24 h, are shown by a heatmap of 13 groups (n = 6) (Fig. 3d). In the heatmap, red represents the model group and green represents the control group. Moreover, 1 represents 23:00–1:00, 2 represents 1:00–3:00, 12 represents 21:00–23:00, and 13 represents the time blood collection was started, 9:00–11:00. A deep red colour represents a high content, and a dark blue colour represents a low content. Detailed information describing the 10 metabolic biomarkers with rhythmic disorder in the control and model groups is shown in Table 2.

**Table 2:** 10 metabolic biomarkers of rhythmic disorder in control and model groups

No.	Name	Formula	Retention time	[M + H]+	[M − H]-	p-value (*m/z*)	VIP	Max (min) peak area/time (control)		Max (min) peak area/time (model)		Metabolic pathway
1	Ornithine	C_5_H_12_N_2_O_2_	0.626	133.0971	–	1.98E−06	1.684	1,548,032,838	9:00–11:00	2,009,357,548	21:00–23:00	Urea cycle, arginine and proline metabolism
2	Glutamine	C_5_H_9_NO_4_	0.768	148.0604	–	4.91E−07	1.580	215,800,083.4	9:00–11:00	313,690,052.1	13:00–15:00	Urea cycle
3	Indoleacetic acid	C_10_H_9_NO_2_	5.837	176.0706	–	1.95E−10	1.778	39,250,248.65	5:00–7:00	139,699,738.5	13:00–15:00	Tryptophan metabolism
4	Citrulline	C_6_H_13_N_3_O_3_	0.761	176.1029	–	1.28E−13	1.421	39,030,378.97	17:00–19:00	62,299,687.79	5:00–7:00	Urea cycle, arginine and proline metabolism
5	Serotonin	C_10_H_12_N_2_O	1.331	177.1022	–	7.58E−14	1.616	250,880,110.6	9:00–11:00	436,645,654.4	11:00–13:00	Tryptophan metabolism
6	l-Kynurenine	C_10_H_12_N_2_O_3_	1.728	209.0920	–	1.91E−08	1.740	68,395,431.46	7:00–9:00	36,004,712.74	11:00–13:00	Tryptophan metabolism
7	d-Proline	C_5_H_9_NO_2_	0.782	116.0706	–	0.002	1.262	847,981,624	17:00–19:00	964,359,024.5	21:00–23:00	Arginine and proline metabolism
8	2-Oxoglutaric acid	C_5_H_6_O_5_	0.796	–	145.0142	1.66E−06	1.439	70,400,138.95	17:00–19:00	242,316,284.8	19:00–21:00	Urea cycle, arginine and proline metabolism, tryptophan metabolism
9	Creatine	C_4_H_9_N_3_O_2_	0.746	132.0767	–	5.88E−09	1.519	21,817,421,950	9:00–11:00	27,898,769,673	21:00–23:00	Arginine and proline metabolism
10	5-Hydroxytryptophan	C_11_H_12_N_2_O_3_	3.14	221.0920	–	1.76E−12	1.419	43,687,663.24	19:00–21:00	32,068,942.09	15:00–17:00	Tryptophan metabolism

**Fig. 4:**
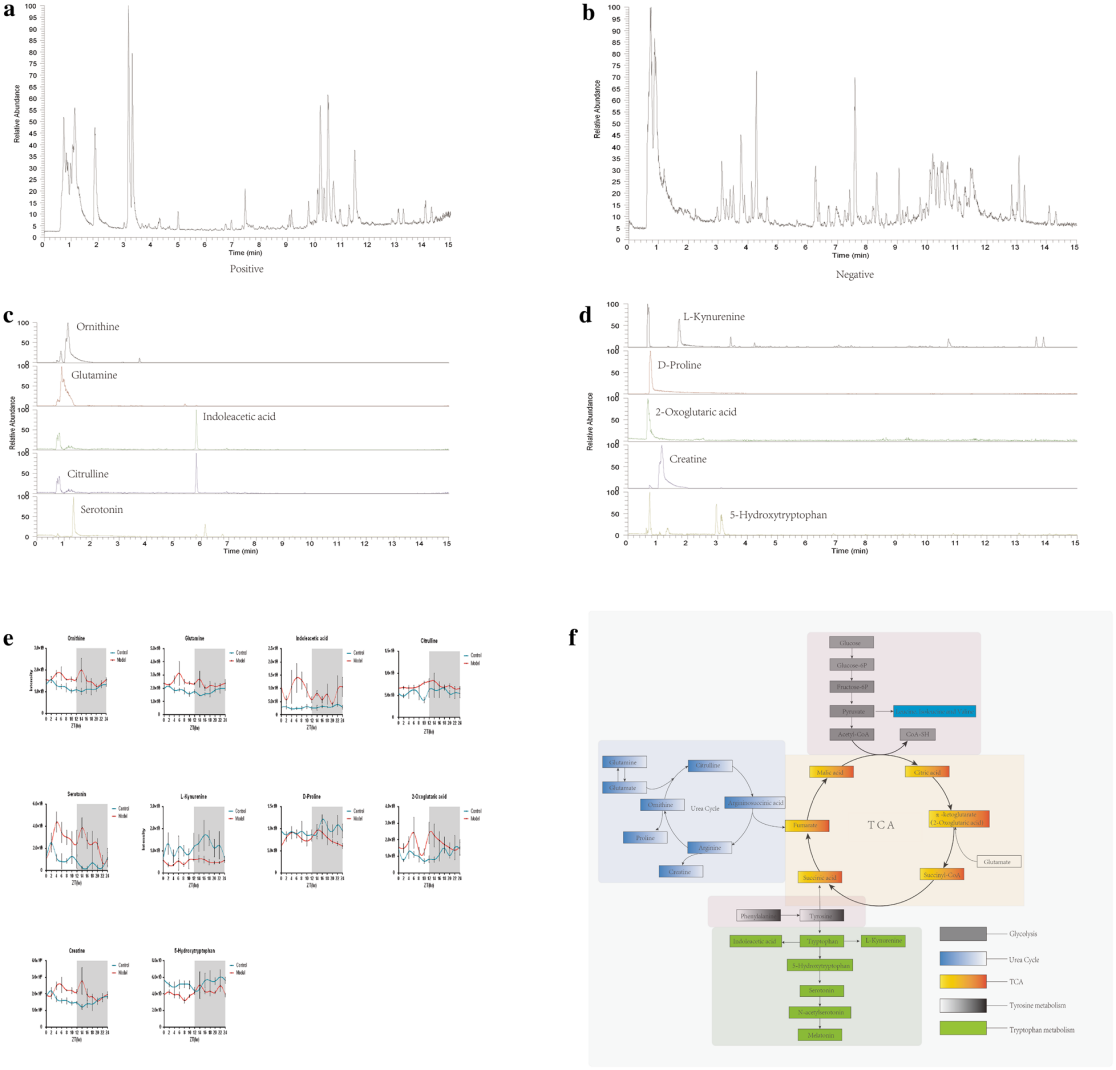
Total ion chromatogram of serum was collected by mass spectrometry under positive and negative ion modes (**a**: positive; **b**: negative). Amino acids were mostly collected in the positive ion mode. **c**, **d** The extracted ion currents of 10 markers, and most substances peaked within 5 min. **e**, **f** The 24-h trend charts of 10 metabolites and metabolic pathways. The amplitude and expression of the model group and the control group are different at 24 h, in which ornithine, citrulline, creatine and 5-hydroxytryptophan have opposite trends. Zeitgeber time (ZT) 0 represents lights on. The metabolic pathways included glycolysis, urea metabolism, the TCA, tyrosine metabolism, and tryptophan metabolism

Total ion chromatograms of serum samples were collected by mass spectrometry under the positive and negative ion modes (Fig. 4a: positive; Fig. 4b: negative). The extracted ion chromatogram currents of 10 markers are also shown in Fig. 4c, d. To clearly observe the 24-h change trend of metabolites, the trends of 10 metabolites and their metabolic pathways were mapped, as shown in Fig. 4e, f. The figure shows that the expression patterns of the model group and the control group are different at 24 h, in particular, ornithine, citrulline, creatine and 5-hydroxytryptophan have opposite patterns.

### Amino acid and their metabolite contents in serum samples

**Fig. 5:**
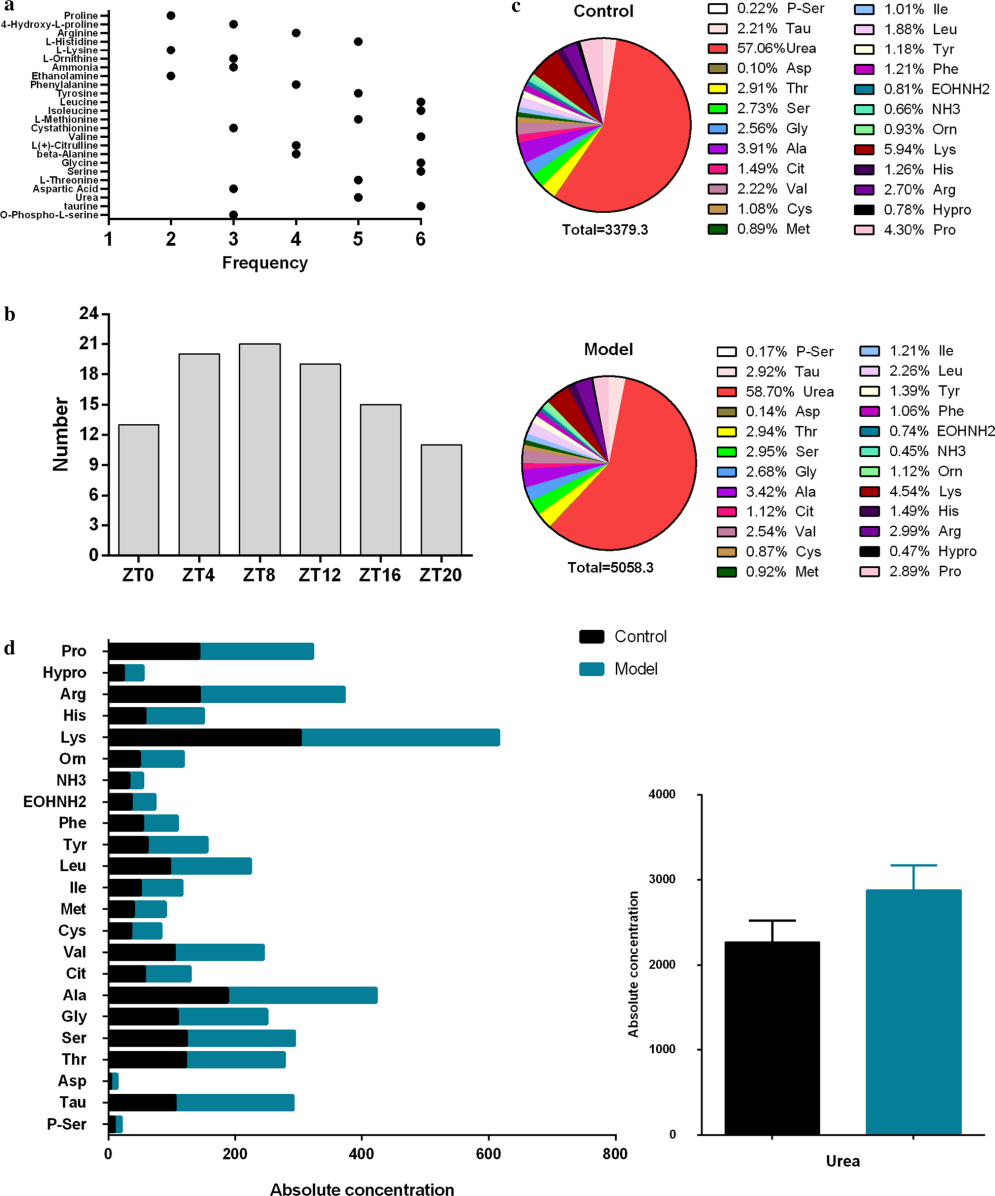
**a** The frequency of differences in the amino acids and their metabolites detected at 6 time points in a day, and **b** the number of amino acids that differed at the different detection times during the day. **c** The ratio of each amino acid/metabolite to the total amino acids and their metabolites in the serum. Different amino acids and their metabolites are identified by different colours. Higher total amino acid content in the serum, higher urea content in the serum, and lower aspartic acid content was observed in the model group than in the control group. **d** The absolute concentration of amino acids and their metabolites at ZT8

Untargeted metabolomics analysis showed that the amino acid and their metabolite contents and the inter-day variation varied greatly between these two groups. Thus, the amino acids and their metabolites in serum were further quantified by an amino acid analyser. The name, abbreviation, formula, and concentrations of the amino acids and their metabolites detected are shown in Table 3. As shown in Table 3, the number of differential amino acids and their metabolites detected at the six time points over 1 day was different, and 15 amino acids and their metabolites had more than 4 time points differences in 24 h (Fig. 5a), accounting for 62.5% of the total number of detected amino acids and their metabolites;. At the ZT4, ZT8, and ZT12 time periods, these two groups showed the largest number of differential amino acids and their metabolites (Fig. 5b). Figure 5c shows the ratio of each amino acid/metabolite to the total amino acids, and Fig. 5d shows the absolute concentration of amino acids and their metabolites at ZT8.

**Table 3:** The concentrations of amino acids and their metabolites in control and model groups

No	Name	Abbreviation	Formula	Concentration/ng/20 µL					
				ZT0		ZT4		ZT8	
				Control	Model	Control	Model	Control	Model
1	O-phospho-l-serine	P-Ser	C_3_H_8_NO_6_P	7.53 ± 1.28	8.77 ± 0.56	9.18 ± 1.02	10.54 ± 0.8*	6.99 ± 0.72	7.82 ± 0.75
2	Taurine	Tau	C_2_H_7_NO_3_S	89.4 ± 14.87	156.16 ± 29.23**	105.62 ± 12.52	185.32 ± 53.88*	63.45 ± 12.86	118.08 ± 10.4**
3	Urea	Urea	CH_4_N_2_O	1993.58 ± 280.6	3181.64 ± 501.01**	2270.86 ± 249.24	2875.2 ± 296.76**	1443.99 ± 181.69	2733.1 ± 669.73**
4	Aspartic acid	Asp	C_4_H_7_NO_4_	2.83 ± 0.97	6.48 ± 6.84	4.74 ± 1.03	15.07 ± 8.03**	3.76 ± 1.17	8.39 ± 0.59**
5	l-Threonine	Thr	C_4_H_9_NO_3_	109.36 ± 26.32	148.87 ± 2.96**	121.97 ± 25.43	155.03 ± 8.01*	71.35 ± 10.6	116.6 ± 8.37**
6	Serine	Ser	C_3_H_7_NO_3_	95.47 ± 23.79	144.88 ± 16.41**	123.28 ± 24.26	170.17 ± 5.26**	74.35 ± 9.42	140.79 ± 24.99**
7	Glycine	Gly	C_2_H_5_NO_2_	91.39 ± 25.25	138.56 ± 27.34*	108.99 ± 14.9	140.77 ± 9.84**	66.44 ± 7.16	132.32 ± 13.29**
8	beta-Alanine	Ala	C_3_H_7_NO_2_	121.32 ± 23.3	174.18 ± 27.67**	187.86 ± 38.23	234.73 ± 15.76*	125.61 ± 23.49	157.25 ± 19.44*
9	l(+)-Citrulline	Cit	C_6_H_13_N_3_O_3_	50.63 ± 9.99	47.95 ± 7.57	57.64 ± 10.22	71.43 ± 9.61*	36.84 ± 7.03	45.7 ± 5.67*
10	Valine	Val	C_5_H_11_NO_2_	79.59 ± 13.24	140.78 ± 12.19**	104.48 ± 14.53	139.69 ± 12.85**	61.31 ± 7.34	106.94 ± 7.88**
11	Cystathionine	Cys	C_7_H_14_N_2_O_4_S	38.01 ± 11.12	50.41 ± 15.56	35.16 ± 8.65	47.93 ± 8.13*	31.54 ± 4.04	46.81 ± 7.8**
12	l-Methionine	Met	C_5_H_11_NO_2_S	31.49 ± 6.36	47.75 ± 3.05*	39 ± 5.84	51.01 ± 2.63**	23.03 ± 2.68	40.86 ± 3.06**
13	Isoleucine	Ile	C_6_H_13_NO_2_	36.31 ± 6.4	69.89 ± 9.12**	51.01 ± 8.3	65.38 ± 5.87*	28.68 ± 3.79	49.32 ± 3.52**
14	Leucine	Leu	C_6_H_13_NO_2_	66.86 ± 13.04	123.81 ± 8.72**	96.98 ± 14.18	126.69 ± 10.73**	52.23 ± 7.05	97.77 ± 8.24**
15	Tyrosine	Tyr	C_9_H_11_NO_3_	48.99 ± 10.45	86.63 ± 7.77**	61.85 ± 8.01	93.72 ± 8.26**	30.17 ± 6.36	59.57 ± 2.58**
16	Phenylalanine	Phe	C_9_H_11_NO_2_	40.07 ± 10.71	55.39 ± 4.23*	54.47 ± 7.87	54.33 ± 3.16	32.52 ± 2.66	45.65 ± 2.73**
17	Ethanolamine	EOHNH2	C_2_H_7_NO	31.14 ± 6.25	37.51 ± 2.25	36 ± 4.9	37.75 ± 1.73	19.6 ± 3.6	30.29 ± 3.43**
18	Ammonia	NH3	NH_3_	21.31 ± 4.22	20.96 ± 1.28	31.9 ± 2.25	22.71 ± 1.07**	14.58 ± 2.7	20.99 ± 6.01*
19	l-Ornithine	Orn	C_5_H_12_N_2_O_2_	25.2 ± 7.31	55.59 ± 26.48	49.03 ± 16.49	69.61 ± 24.31	26.53 ± 7.93	46.35 ± 13.06*
20	l-Lysine	Lys	C_6_H_14_N_2_O_2_	177.08 ± 49.69	242.23 ± 34.89*	302.61 ± 69.01	312.84 ± 21.66	175.9 ± 25.65	224.72 ± 18.64**
21	l-Histidine	His	C_6_H_9_N_3_O_2_	39.06 ± 11.15	73.36 ± 5.81**	58.14 ± 12.38	92.04 ± 5.41**	33.11 ± 3.24	69.15 ± 9.37**
22	Arginine	Arg	C_6_H_14_N_4_O_2_	87.69 ± 29.44	130.39 ± 34.45	143.46 ± 47.1	228.83 ± 45.41*	83.09 ± 22.36	159.51 ± 18.75**
23	4-Hydroxy-l-proline	Hypro	C_5_H_9_NO_3_	17.3 ± 4.86	21.81 ± 3.51	23.53 ± 4.75	31.29 ± 1.5*	28.78 ± 4.75	26.8 ± 2.1
24	Proline	Pro	C_5_H_9_NO_2_	123.73 ± 24	134.37 ± 35.81	142.67 ± 31.36	179.19 ± 13.8*	135.99 ± 29.41	120.27 ± 22.2

No	Name	Abbreviation	Formula	Concentration/ng/20 µL					
				ZT12		ZT16		ZT20	
				Control	Model	Control	Model	Control	Model
1	O-phospho-l-serine	P-Ser	C_3_H_8_NO_6_P	6.09 ± 0.4	7.57 ± 0.29**	7.49 ± 0.9	9.06 ± 0.76*	7.7 ± 1.33	7.68 ± 0.51
2	Taurine	Tau	C_2_H_7_NO_3_S	52.71 ± 7.5	95.58 ± 29.03*	75.61 ± 19.82	191.56 ± 56.4**	88.45 ± 37.02	136.85 ± 22.83*
3	Urea	Urea	CH_4_N_2_O	1643.64 ± 257.53	2518.04 ± 233	2507.3 ± 462.32	3523.42 ± 675.16*	2393.68 ± 230.25	2892.78 ± 216.13*
4	Aspartic acid	Asp	C_4_H_7_NO_4_	2.37 ± 0.86	4.04 ± 4	3.51 ± 0.74	5.99 ± 5.02	3.72 ± 1.48	9.11 ± 2.46*
5	l-Threonine	Thr	C_4_H_9_NO_3_	68.13 ± 8.72	134.55 ± 15.87**	133.06 ± 23.01	193.15 ± 32.08**	133.96 ± 26.15	144.05 ± 15.28
6	Serine	Ser	C_3_H_7_NO_3_	68.98 ± 9.34	136.75 ± 17.07**	117.15 ± 23.54	172.09 ± 18.44**	108.03 ± 10.88	130.9 ± 6.82**
7	Glycine	Gly	C_2_H_5_NO_2_	66.05 ± 8.43	127.28 ± 25.53**	114.36 ± 17.37	146.73 ± 12.3**	106.06 ± 14.38	125.89 ± 6.29*
8	beta-Alanine	Ala	C_3_H_7_NO_2_	93.75 ± 20.84	131.79 ± 10.98**	143.49 ± 30.66	189.73 ± 41.22	151.78 ± 58.34	152.69 ± 17
9	l(+)-Citrulline	Cit	C_6_H_13_N_3_O_3_	40.17 ± 7.29	55.04 ± 11.06**	68.99 ± 11.24	71.19 ± 9.41	69.23 ± 14.81	52.05 ± 8.39*
10	Valine	Val	C_5_H_11_NO_2_	52.87 ± 5.59	106.9 ± 3.65**	89.59 ± 15.55	151.47 ± 7.11**	91.22 ± 18.03	123.81 ± 18.32*
11	Cystathionine	Cys	C_7_H_14_N_2_O_4_S	34.76 ± 4.3	11.07 ± 1.72**	50.9 ± 16.54	57.42 ± 12.81	34.06 ± 16.77	45.99 ± 9.01
12	l-Methionine	Met	C_5_H_11_NO_2_S	22.91 ± 2.73	40.5 ± 2.83**	38.06 ± 8.22	56.63 ± 6.95**	38.56 ± 8.62	42.59 ± 2.62
13	Isoleucine	Ile	C_6_H_13_NO_2_	22.89 ± 3.29	48.25 ± 2.12**	40.26 ± 9.27	73.56 ± 3.86**	38.95 ± 8.6	59.34 ± 11.08**
14	Leucine	Leu	C_6_H_13_NO_2_	41.1 ± 6	91.79 ± 3.38**	75.7 ± 15.17	135.66 ± 7.29**	74.04 ± 17.26	109.66 ± 18.72**
15	Tyrosine	Tyr	C_9_H_11_NO_3_	25.66 ± 3.98	46.96 ± 9.19**	41.95 ± 7.72	67.78 ± 6.33**	51.25 ± 22.26	68.33 ± 12.43
16	Phenylalanine	Phe	C_9_H_11_NO_2_	31.4 ± 2.43	48.76 ± 2.51**	50.56 ± 7.6	65.59 ± 6.27**	50.68 ± 14.25	51.38 ± 4.87
17	Ethanolamine	EOHNH2	C_2_H_7_NO	19.3 ± 4.73	37.32 ± 3.23**	34.33 ± 3.76	43.61 ± 9.42	37.24 ± 9.26	37.71 ± 2.92
18	Ammonia	NH3	NH_3_	19.28 ± 13.34	21.92 ± 0.9	28.19 ± 1.57	27.45 ± 2.43	28.29 ± 1.48	23.15 ± 1.32**
19	l-Ornithine	Orn	C_5_H_12_N_2_O_2_	23.43 ± 5.09	42.01 ± 6.8**	37.49 ± 13.02	59.26 ± 10.63*	35.61 ± 11.29	49.12 ± 14.99
20	l-Lysine	Lys	C_6_H_14_N_2_O_2_	155.67 ± 22.76	179.67 ± 21.54	221.42 ± 29.33	243.95 ± 14.51	221.59 ± 63.78	180.88 ± 9.13
21	l-Histidine	His	C_6_H_9_N_3_O_2_	31.39 ± 2.34	64.71 ± 2.26**	54.53 ± 9.43	87.35 ± 9.32**	55.3 ± 13.83	66.53 ± 6.2
22	Arginine	Arg	C_6_H_14_N_4_O_2_	74.32 ± 16.8	130.84 ± 12.62**	86.02 ± 15.09	151.18 ± 23.29**	90.94 ± 39.81	117.64 ± 9.45
23	4-Hydroxy-l-proline	Hypro	C_5_H_9_NO_3_	30.73 ± 4.26	21.38 ± 6.01*	26.25 ± 6.19	24.48 ± 4.15	23.97 ± 4.23	18.92 ± 2.65*
24	Proline	Pro	C_5_H_9_NO_2_	132.18 ± 19.35	139.03 ± 19.59	175.92 ± 43.29	190.65 ± 44.34	175.39 ± 43.84	118.41 ± 22.13*

** *p *< 0.01 model group compared with control group

* *p *< 0.05 model group compared with control group

## Discussion

Our study used an untargeted metabolomics approach to demonstrate the 24 h metabolic profile of the SHR group and the WKY control group. SHRs are a well-known model of essential hypertension [15]. An important feature of the SHR model is that it was originally derived from the normal WKY strain [16]. Therefore, in basic scientific research on the level of hypertension animals, WKYs are used as a normal control group for SHRs [2, 17–19]. Metabolomics platforms have been used to measure and identify key biomarkers related to pathological conditions. In recent years, a series of advances have been made in the combined analysis techniques of metabolomics, especially LC–MS and nuclear magnetic resonance (NMR) [20, 21]. Circadian rhythm research also relies on these platforms, as metabolomics can reflect the whole physiological state by the detection of specific metabolites [6, 9, 14]. These high-throughput, high-sensitivity and high-resolution research platforms have promoted a series of studies on circadian rhythm metabolism.

In this study, the 24-h rhythms of serum metabolites in rats were characterized by using LC–MS metabolomics, including arginine, proline, serotonin, phenylalanine, citric acid, L-malic acid and succinic acid, etc., offering novel insight into hypertension. In addition, based on the untargeted metabolomics outcomes the concentrations of amino acids and their metabolites were determined, and the changes in leucine, isoleucine, valine, taurine, serine, and glycine were the most obvious. In the current hypertension-circadian rhythm study, the metabolic profile of SHRs was found to be different from that of WKYs from a holistic perspective. The mass spectral intensity of 112 metabolites varied at different time points, indicating that the concentration of metabolites constantly changes throughout the day. Multivariate statistical analysis was used to identify differential metabolites between the control and model groups and rhythmic biomarkers. The results from this study suggested that the circadian rhythm of hypertension is closely related to the urea cycle, arginine and proline metabolism and tryptophan metabolism. Interestingly, the disruption of metabolic pathways that occurs in many metabolic disorders has been reported to impact gene expression programmes in tissues and organ physiology by affecting molecular clock rhythms [22, 23].

The metabolic pathways that link hypertension with circadian rhythm are not well understood. Metabolic pathway enrichment analysis revealed that the urea cycle, which includes ornithine, purine, glutamic acid, citrulline and 2-oxoglutaric acid, was the most significantly impacted metabolic pathway. Notably, the urea cycle also known as the ornithine cycle, is the final pathway in the removal of surplus nitrogen from the body, as well as the major route for the detoxification of ammonia [24]. As energy is required to dispose of excess ammonia, it is apparent that ATP must be available for the urea cycle to properly function. Thus, the urea cycle is closely linked to the citric acid cycle, which derives one of its nitrogens from the transamination of oxaloacetate to form aspartate and returns fumarate to the cycle [25, 26].It is reported that an LC–MS method was used to quantify amino acids to investigate urea cycle disorders. It discovered that urea cycle disorder not only disturbed the metabolism of amino acids involved in the urea cycle and induced the accumulation of ammonia detoxification, but also interfered with the metabolism of amino acids related to some nervous systems, such as pipecolic acid and N-acetylaspartic acid [27]. Our research was consistent with this study. During the development of hypertension, the contents of amino acids such as ornithine, glutamic acid and citrulline involved in the urea cycle were higher in the hypertension group than in the control group, and the urea cycle reaction substrates were abundant, resulting in an increase in blood ammonia. In addition, arginase, the enzyme that converts arginine to ornithine and urea, competes with oxide synthetase (NOS) for arginine leading to a decrease in nitric oxide (NO) expression and the destruction of the urea cycle. As NO is a critical factor in cell growth and vasodilation, and the decrease of in NO leads to increased hypertension [28, 29]. The 24-h metabolite rhythms in overweight/obese individuals and in individuals with type 2 diabetes showed that proline has robust daily rhythms and that isoleucine and valine rhythms were disturbed in patients with type 2 diabetes [6, 9]. Our animal experiments also found similar results. The serum content of branched-chain amino acids (BCAAs) was significantly higher in the hypertension groups compared with the control groups, which indicated that animal experiments could reflect the changes in the human body to a certain extent. BCAAs may affect cell signalling and aggravate oxidative stress [30], which contributes to endothelial cell damage. Endothelial cell damage is one of the causative factors of hypertension [31]. Therefore, in addition to cell signalling and oxidative stress, high concentrations of BCAAs are commonly associated with hypertension. These results also indicated the role of the circadian metabolic processes in hypertension.

Our pathway analysis revealed that arginine and proline metabolism were enriched in hypertension. Both in vivo and in vitro experiments have demonstrated that amino acids are circadian [32]. In general, amino acids rarely remain free in cells, because they have many physiological functions, such as (i) protein synthesis in a fed state; (ii) gluconeogenesis during fasting; (iii) metabolism into bioactive molecules; or (iv) degradation to liberate ammonia, which is fed into the urea cycle [33]. In a randomized, double-blind, crossover trial investigating air purification, 10 amino acids (arginine, leucine, histamine, threonine, serine, glutamine, lysine, phenylalanine, tyrosine, and tryptophan) were also clearly associated with PM2.5, which has been proven to be related to oxidative stress, which is associated with elevated blood pressure [34]. l-Histidine, l-phenylalanine, l-arginine, l-glutamine, l-valine, l-isoleucine, l-leucine, l-tyrosine, l-proline, and l-glutamic acid showed rhythm disturbances in hypertension based on the metabolomics method. In addition to amino acids, metabolites in the TCA such as succinic acid, L-malic acid, 2-oxoglutaric acid, and citric acid, and glucose also had abnormal rhythms and contents in hypertension. These results showed that amino acids may affect energy metabolism by increasing metabolic rate and energy expenditure to meet the physical needs in hypertensive patients with TCA inhibition. Moreover, an increase in arginine and proline metabolism is closely related to arterial injury. As mentioned above, arginase and NOS competitively bind to arginine to produce NO and ornithine (the precursor of polyamines and proline), respectively [35]. An increase in polyamine synthesis has been observed in response to the proliferation of vascular smooth muscle cells and the development of intimal thickening [36], which could also contribute to hypertension. Moreover, it has been hypothesized that amino acids change significantly in the circadian misalignment individuals with of type 2 diabetes, prediabetes/obesity, reduced insulin sensitivity and impaired glucose homeostasis and those consuming a high-fat diet [37, 38]. This hypothesis was also confirmed in our study on hypertension rhythm, indicating that changes in amino acid contents are an independent risk factor for cardiovascular disease.

It has been reported that amino acids in human plasma, including tryptophan, tyrosine, phenylalanine, methionine, cysteine, glutathione and homocysteine have circadian rhythms [39–42]. Because amino acids can be used for gluconeogenesis under energy deprivation [43], rhythm disorders in the catabolism of amino acids, especially BCAAs, including leucine, isoleucine, and valine, can lead to serious cardiovascular diseases [44]. Serum amino acids are markers of prediabetes, insulin resistance and incident diabetes [45]. Abnormal amino acid metabolism was also observed in hypertension. The quantitative analysis of amino acids revealed that at least one amino acid was significantly different at the same time and each amino acid had at least one time difference within a day. Circadian clocks are fundamental physiological regulators of energy homeostasis. For instance, the loss of bmal1, a circadian clock gene, is associated with the accumulation of amino acids [46]. Accordingly, amino acid contents can reflect the expression of circadian clock genes. In this study, the accumulation of BCAAs was found by amino acid quantification, which revealed that hypertension causes metabolic deficiencies in BCAAs. Additionally, serum amino acids were consistently higher in the hypertension group than in the control group over 24 h. High concentrations of BCAAs promote oxidative stress, inflammation and migration via mTORC1 activation [47]. In addition, leucine, a branched-chain amino acid, can activate mTORC1 signalling to increase sympathetic nerve activity and arterial pressure in anaesthetized rats [48–50]. Therefore, it is speculated that BCAAs are also mainly related to elevated blood pressure through the mTORC1 pathway.

Tryptophan metabolism also showed an oscillatory imbalance in hypertension except for the urea cycle and arginine, proline and BCAA metabolism. In the tryptophan metabolism pathway, the concentrations of indoleacetic acid, serotonin, and 2-oxoglutaric acid increased, while the concentrations of l-kynurenine and 5-hydroxytryptophan decreased. Interestingly, kynurenine, a marker of renal function, was also altered by prior exposure to simulated night-shift work [6, 51]. It has been reported that kynurenine serves as a negative feedback regulator in the event of increased vascular pressure. In arteries, kynurenine may cause arterial relaxation mainly via the cAMP pathway, ameliorating the constantly increased wall stress [52]. However, a continuous increase in arterial blood pressure and a decrease in kynurenine expression indicate that the steady state of tryptophan metabolism is destroyed and the feedback loop is out of order, which in turn leads to an increase in blood pressure. Hypertension patients may have suppressed secretion of melatonin, a hormone that regulates the circadian cycle and has multiple cardioprotective properties [53]. Additionally, melatonin has been discovered to have antihypertensive effects, most likely because of its anti-inflammatory and reactive oxygen species scavenging properties [54]. 5-Hydroxytryptophan produces serotonin under the action of tryptophan decarboxylase. Through additional reactions, serotonin can produce N-acetylserotonin, the precursor of melatonin under the action of serotonin acetylase [34]. Our study showed that serotonin levels increase but that melatonin levels decrease in hypertension. This finding may indicate that changes in the content or activity of enzymes occur during the production of downstream metabolites of serotonin, which may be helpful to elucidate the pathogenesis of hypertension.

Compared with SHRs, human hypertension developed with many risk factors, including ethnicity, familial, dyslipidemia, and lifestyle [55]. Base on this, hypertension patients generally accompany with other co-morbidities, such as obesity, diabetes, left ventricular hypertrophy, albuminuria and renal dysfunction [56]. Although there may be some differences in the results of the experiments translating into humans, the pathogenesis of human hypertension is approximately similar to that of SHRs. The common mechanism of vascular injury in hypertension patients and SHRs including endothelial dysfunction, atherosclerosisi, and is associated with an imbalance of vascular NO bioavailability and oxidative stress [57]. Moreover, compared with hypertension patients, the metabolic pathways of SHRs to some extent show similarities. For instance, in the study of metabolomics on human hypertension, it found that the differential biomarkers are closely related to amino acid metabolism [58], and the study of young hypertensive men on metabolomics also indicate that variances of amino acids have a major impact on the metabolic change [59]. In addition, metabolites variability within a 24 h period has been closely associated with metabolic disorders [6, 9]. Therefore, the circadian metabolic phenotype strategy of our study would be translated and explained the mechanism of metabolic rhythm of hypertension patients.

Our study also has several limitations. The central circadian clock genes and the peripheral circadian clock genes were not detected, so the relationship between metabolites and genes was not elucidated. However, it has been reported that the loss of the endogenous circadian clock can lead to the destruction of metabolic homeostasis [60]. Temporal profiles may be controlled by the SCN clock [61]. Although, it is currently understood that the circadian rhythm is regulated by negative feedback loops to ensure that cells, organisms and metabolites are consistent with the light–dark cycle, important questions remain unknown regarding the biophysical mechanisms that underlie transcriptional oscillations and the regulation of metabolites [62, 63].

## Conclusion

The mechanism of hypertension in general physiology and pathology has been widely studied, and a large amount of knowledge has been gathered in the study of antihypertensive drugs. Nevertheless, the effect of circadian rhythm on metabolites is also not negligible. Under the guidance of chronobiology and timing-based administration, the study of the dynamics of hypertension is particularly important for the interpretation of the mechanism. From this study, metabolite concentrations constantly change throughout the day, and the amplitude and phase of these changes that occur in hypertension deviate. These rhythmic indicators should also be included in future research and treatment. In this study, the results not only showed that the metabolic profiles of SHRs were different from those of the control group, but also revealed that the endogenous metabolites in SHRs have different characteristics. In particular, the 24-hour oscillation patterns of ornithine, citrulline, creatine and 5-hydroxytryptophan in the hypertension group were different from those in the control group, and BCAAs accumulated in hypertension. These findings provide good further research directions and may help to reveal the pathology of hypertension.

## Data Availability

Not applicable.

## Abbreviations

TCA: tricarboxylic acid cycle
SCN: suprachiasmatic nucleus
LC–MS: liquid chromatography–mass spectrometry
NMR: nuclear magnetic resonance
SHRs: spontaneously hypertensive rats
WKY: Wistar-Kyoto Rats
QC: quality control
CD: compound Discoverer
PCA: principal component analysis
PLS-DA: partial least-square analysis
BCAAs: branched-chain amino acids
NOS: nitric oxide synthase
NO: nitric oxide
